# Dr. Blake on Monomania

**Published:** 1838-01-01

**Authors:** 


### Dr. BLAKE ON MONOMANIA.

Dr. Blake is well known to the profession by his original and standard work on Delirium Tremens, as well as by several other valuable publications. As physician to the Lunatic Asylum near Nottingham, he has of late years had excellent opportunities of studying insanity in all its varied forms—opportunities which, it is evident, he has not failed to turn to good account. The occasion of his present appearance before the public is as creditable to his humanity, as the manner of it is to his knowledge of mental disease.

At the last Summer Assizes for the County of Nottingham, a man named Greensmith was tried for the murder of his four children, whom he had strangled a few months previously. Between the perpetration of the unnatural crime and the period of his trial, Dr. Blake had three times (at long intervals) visited the prisoner in his confinement, and had satisfied himself that he was labouring under melancholic monomania, and that he had perpetrated the crime under the delusion, that it was better his children should die by his hands, and that he himself should suffer the utmost penalty of the law, than that he and they should be reduced to want. Strongly impressed with the conviction above-mentioned, Dr. Blake attended the trial as a witness, and the following is a brief summary of his evidence. He deposed, "that he had visited the prisoner on three several occasions, viz. at the time of his commitment, and twice since; the last time was about a fortnight ago. He had been in Court ever since the evidence of the surgeon had been tendered, but not before. His visits to the prison were in consequence of its being rumoured that he was insane; and as physician to an asylum for the reception and cure of such parties, he felt it to be his duty towards the prisoner, and towards society, to ascertain how far that rumour was founded on fact. With respect to the conduct of the prisoner, the most minute, and in fact the only account, was to be found in the prisoner's own deposition. That deposition he at first attentively perused, and he was of opinion from its statements, that the prisoner laboured under a *delusion*, and that the act arose from *that delusion*. On visiting the prisoner, he was confirmed in this opinion, and could corroborate the facts deposed by Mr. Attenburrow.* The prisoner, when questioned, had admitted that he had a sister, who had been, some time since, under the care of witness for lunacy and mental derangement. Witness was not aware of this, nor did he remember it at the time, but on examining the books of the institution, he found that it was so, and that she had laboured under the same kind of impulse to destroy life, &c. &c. She is now a cook, employed occasionally in families: she came to the Asylum five years ago; and he had *understood* that her grandmother laboured under a similar infirmity." (The learned Judge objected to this part of Dr. Blake's evidence, as being equally remote from fact, or medical opinion founded on fact.)

*By the Counsel for the prosecution.*—"It appeared to him that the prisoner has laboured under a delusion of the imagination, that it was better for him, and for his family, that he should destroy his children and be executed for the act, than let them go to the workhouse."

* This gentleman stated, "that when he visited the prisoner, as surgeon of the county jail, he appeared composed and unmoved by any allusion to the position in which he was placed, by the commission of the crime with which he was charged; that his pulse was slow, being seldom above 60 in a minute; that the tongue was white and thinly furred, as may be remarked in almost all melancholic patients, and that he slept but little at night; while his appetite for food was exceedingly good, such as insane persons often evince, notwithstanding the foul appearance of the tongue, and the sluggish state of the circulation." To this account of symptoms Dr. Blake adds, "the peculiar monomaniacal cast of eye was strongly apparent in the prisoner's looks, and when I spoke to him of his execution being the natural consequence of his crime, he answered without emotion, that he expected nothing else, while the pulse on which I kept my finger, did not vary a single beat in a minute; but on the contrary, when I asked if he had not been attached to his children, he evinced immediate and considerable nervous agitation, and shed tears abundantly."

On the foregoing evidence Mr. Justice Park (who tried the case) commented as follows :—"The physician who had stept forward to urge the plea of insanity on behalf of the prisoner, had stated a doctrine very opposite to this," (the Judge alluded to Sir Matthew Hale's definition of criminal responsibility) "and one which was altogether inadmissible in a court of justice: and which gave him some surprise, when he heard it advanced by a Gentleman, occupying so important a station in society. Nothing could be more contrary to law, than to infer insanity from the very malignity and atrocity of the crime. It was true, that such crimes could never be committed by men who were in possession and control of a right reason and a proper mind; but it was his duty to inform the jury, that the complete possession of reason was not essential to constitute the legal, any more than the moral responsibility of any man; it being merely necessary that the party should have sufficient knowledge and reason to discriminate between *right* and *wrong*."

These remarks were contained in the Judge's address to the Jury, who returned a verdict of "guilty," and the prisoner was in consequence sentenced to undergo the extreme punishment of the law.

But Dr. Blake did not let the matter rest here. He instituted a correspondence on the subject with the Secretary of State, to whom he addressed two letters, which have since been published. They are now before us, and our conviction, after a careful perusal of them, is, that the prisoner was decidedly insane both at the time of committing the crime, and up to the period of his trial. In these letters Dr. Blake disclaims the doctrine attributed to him by the learned Judge, a doctrine which would "infer insanity from the very malignity and atrocity of the crime." He shows that he by no means rested his conclusion upon this *circumstance alone, but upon a union of all the facts connected with the case.* With many of these, however, the Jury unfortunately could not be made acquainted, for the prisoner had taken no care to provide counsel for his defence, and those benevolent individuals, who, believing him insane, took an interest in his case, had erroneously calculated on obtaining, in his behalf, the appointment of a Crown advocate. In consequence of this omission, several witnesses, whose evidence would have been sufficient to circumstantiate Dr. Blake's views, were either not examined, or were examined so imperfectly, that their testimony had not the effect which it otherwise must have had. Some of them could have deposed to the undoubted insanity of the prisoner's grandmother, uncle, and sister, the last of whom, (as Dr. Blake stated in his own evidence, and as might have been legally proved) had felt not only the same kind of impulse to destroy life, but the exactly similar desire to destroy her children as her brother. "The learned judge (continues Dr. Blake, addressing the Secretary of State) objected to my remarks on the similarity of the delusion, and said it was *sufficient* for me to state, that the sister had been confined in an asylum. Your Lordship will observe, no allusion has been made by the Judge to this important fact, namely, the similarity of the delusions of the brother and sister, in his charge to the Jury. Now, my Lord, I do not presume on legal knowledge, but it appeared to me that this coincidence regarding a brother's and sister's delusion, was a circumstance worthy of notice by all those who admit the *hereditary* influence of insanity, and one which, had the prisoner had a Lord Erskine to plead for him, would not, I apprehend, have been passed over in silence."

The learned Judge, after having found fault with the grounds on which I drew my conclusions, is made to say, "that it was his duty to inform the Jury that the complete possession of reason was not essential to constitute the legal, any more than the moral responsibility of any man; it being merely necessary that the party should have sufficient knowledge and reason to discriminate between right and wrong."

I would respectfully quote Lord Erskine's opinion against this. His Lordship's proposition is, "that, to absolve from criminal responsibility, there must, first, be *delusion;* and, secondly, the *delusion* and the *act* must be connected." Now, in my opinion, this was exactly Greensmith's case. He was predisposed by hereditary taint to insanity; and the loss of his wife, and pecuniary distress, brought on the delusion under which he acted.

Lord Erskine, in his celebrated* defence of Hatfield, who attempted George the Third's life, says, "In *all* the cases which have filled Westminster Hall with the most complicated considerations, the lunatics, and other insane persons, who have been the subjects of them, have not only had the most perfect knowledge and recollection of all the relations they stood in towards others, and of the acts and circumstances of their lives, but have in general been remarkable for subtlety and acuteness." This is not the Hon. Judge Park's opinion; but it goes in direct opposition to it to establish, that a man may commit an insane act under a delusion, and consequently be exempt from responsibility, although he may, at the same time, possess memory, and a knowledge of the relations in which he stands towards others, and of the acts and circumstances of his life, and consequently possess reason to discriminate between *right* and *wrong*. This, my Lord, is an axiom too well established in the opinion of persons conversant with the subject of insanity, to require further consideration. Many persons are sane on all but one subject; and that may be a desire to destroy their offspring; while they may be sensible of the enormity of the crime, and yet be incapable of resisting it. There is at present under the surveillance of the governor of the Nottingham workhouse, a female who could scarcely resist the propensity she felt to destroy her children, and only found security from the commission of the crime (*of the enormity of which she felt a due sense*) by absenting herself from her home. We have also, in the Nottingham asylum, a female, who feels such a strong suicidal tendency, that she cannot live out of the asylum, lest she should yield to her feelings, on which she will converse rationally. Writings on insanity abound with examples on this subject, but I only quote cases now existing in Nottingham.

Again, in answer to that part of the learned Justice Parke's charge wherein he says, "The duty of the jury was to inquire and decide whether the prisoner at the bar was able to judge between right and wrong at the time that he committed this horrible deed." I shall offer the well-known case of Jonathan Martin. I saw this deluded man a short time previous to his trial, when I was stationed at York as surgeon to the 7th Dragoon Guards. I visited him in the jail, accompanied by Dr. Wake, the physician to the lunatic asylum. I asked him if he was not aware that he was doing wrong when

* The trial took place before Lord Kenyon, who assented to the principle, as did also Lord Redesdale, who was then the Attorney-General.

he set fire to the Minster, and liable to be hanged for so doing. His answer was, "Yes, according to the law of man, I know I was; but as I had the command of God to do so, He will protect me if He pleases; and if it is His pleasure that I should die, I am ready to comply with the Divine will." I saw him since at Bethlem, and asked him if he recollected seeing me at York. He said, "No; but since you speak of York, perhaps you can tell me if the Minster is restored." On my answering in the affirmative, his remark was, "I hope they will make a better use of it." On every other subject he was perfectly rational, and appeared to possess ten times more intellect than the unfortunate man, whose case I advocate, ever displayed. How did it happen, then, that Judges were found to acquit Martin on the grounds of insanity, if a knowledge of right from wrong was to constitute the test of a man's criminalness or innocence? But the fact is, poor Greensmith had no one to plead for him at his trial. The only kind of question put, in favour of the prisoner, to the witnesses against him, was, "Did you, at any time, perceive that the prisoner evinced symptoms of insanity?" Such questions could not draw from witnesses of the class that were examined appropriate answers on the subject. They who, one and all, stated that the prisoner was a *kind* father and a *sober industrious* man, could see nothing of insanity in his conduct; but had they been asked if he was taciturn or talkative, or melancholy or gay, or was he seen to shed tears without apparent cause, and such like questions, the truth would have come out; and had the prisoner possessed the slightest intelligence necessary for the protection of his life, he would have had witnesses ready, and an advocate appointed, who would have quoted authority to shew the jury, that precedents existed where a prisoner was acquitted who knew right from wrong, although he actually fired at a king!! But, alas! he was totally incapable of any mental exertion. Indeed the probability of his execution formed a part of his delusion, and of course deprived him of mental powers on that particular subject.

To give your Lordship an idea of the disadvantages under which the prisoner laboured by not having counsel, I beg to submit one fact. Mr. Attenburrow, the surgeon of the county jail, wrote two questions, and requested the counsel for the prosecution to put them to a witness, Anne Greensmith. The first question went to ascertain if the prisoner had ever asked the witness to come and stay with him until he procured a housekeeper. This question the counsel put to the witness, but the second question he omitted. Now the second question would have elicited an answer proving the existence of delusion, connected with destructive feelings, so far back as before his last housekeeper came to live with him—a fact which, when properly stated by counsel, must have had due weight with the jury. I deplore the loss of this evidence, because it was essential, and must have been brought forward had he had counsel. But I do not wish to insinuate that the learned counsel for the prosecution suppressed the question intentionally, as he could *not* have known the answer which it would have elicited. I beg also to call your Lordship's notice to that part of the charge where the Judge particularly directed the "attention of the jury to that *act of reason* manifested by the prisoner in stating to Brownrow his motives for keeping away from Basford, and wishing to be denied to all who inquired for him." Here again, I respectfully differ from him. I consider that no sane man would, after having left four children murdered at Basford, caution a man of a near neighbouring village, who knew he was from Basford, to deny him to all who inquired after him. This gleam of self-preserving reason was that which often returns for an instant after the accomplishment of crimes dependant on delusion.

Jonathin Martin ran away, after he set fire to the Minster, and was only taken in the extreme north of England ; and yet he was not condemned. In another part of the Judge's charge, he says : "After destroying two of his children, he went down and sat by the fire considering :"—but what did he consider ?—This the Judge has omitted to state. He considered, whether he might not be capable of maintaining two of them. Was this, even for an instant, the consideration of a sane mind ? How could he expect to be allowed to maintain them ?—reason, aided by the *love of his children*, made an effort to arrest him in his crime ; but his original delusion of destroying the whole, and being executed *to save them from going to the workhouse*, prevailed. "The result of his consideration," the Judge says, "was an opinion and belief that he should suffer what the law allotted, whether he proceeded with his first intention or not, and therefore, that he would go on with it." Although I do not wish to attempt to prove the existence of a delusion in the case from the enormity of the crime alone, yet I think it will be admitted, that if a sane person formed the project of destroying his children, he would not have accomplished his wicked design, in the *manner* in which Greensmith did. The calmness in the manner of this man after the act, on which the Judge lays so much stress, is also in my mind, a proof of his insanity. It is only hardened offenders, accustomed to steep their hands in human blood, or *persons affected with insanity*, who have the power to commit crimes such as this, without evincing any fear or repentance. Now this unfortunate man, as was proved in evidence, was anything but one given to commit cruel crimes ; hence I must, with all due respect, again differ from the learned Judge in his conclusion. Indeed, I differ from him in considering that so atrocious an act, so contrary to the best feelings of human nature, without any one rational motive, without any hope of gain or worldly advantage, without anger, (for he took affectionate leave of his children before he destroyed them, and again, before he finally left them,) could have been perpetrated by an individual so tender in his affection, so sober, and so industrious in his habits, as he was proved to be, if he were not labouring under a delusion !

"In conclusion, (continues Dr. B. in an Appendix to the letters,) I beg it to be well understood, that in venturing to differ from many highly enlightened and humane persons on the question of the propriety of Greensmith's execution, I do so with the greatest deference ; and solely from a moral conviction, arising out of my experience in cases of insanity, together with the evidence which I was enabled to collect relative to the prisoner's state of mind, that he laboured under a delusion when he destroyed his four children. I am fully aware, also, that a number of very just and intelligent individuals entertain the opinion, that such an atrocious crime, ought, if it were only for example sake, to be punished with death :—To this plausible conclusion, the justice of which however cannot be maintained either in law or equity, I would respectfully submit the fact, that a great proportion of those who are guilty of infanticide, either attempt to destroy themselves immediately, or look forward to dying by the hands of justice. Thus their own death almost invariably forms part and parcel of the delusion under which they act ; and by their execution, you would, generally speaking, only accede to an ardent desire, felt by them at the moment of the commission of their crime. On the other hand, as mono-maniacs possess the power of reasoning, more or less soundly, on all subjects, save that on which they are insane, and even on that point they can sometimes reason plausibly to a certain extent, their condemnation to seclusion, by thwarting a principal part of their delusion, namely, the desire they may feel to be executed, must be much more likely to deter others from committing such crimes, than the prospect of death, the infliction of which, as I have already observed, is only the consummation of, perhaps, the main object of their delusion."

We have great pleasure in laying before our readers the foregoing clear and just remarks on a form of insanity, which, interesting in itself, is peculiarly so when it comes to bear on the question of moral responsibility. It is often the duty of the members of our profession, aided by the light of science, to pursue wickedness through the mazy labyrinth which her cunning had devised—to trace out, as it were with senses denied to other men, the most secret crimes, and by bringing them home to the astonished perpetrators, convince them, that since solitude and darkness cannot skreen them from human detection, they are but a poor defence against the eye of an all-seeing God. But Justice, armed with her sword, is only half armed; her protecting shield is an equally important part of her equipment. Hence, while it is often the peculiar duty of the medical practitioner to bring the criminal to punishment, it is also his high privilege (and he ought ever to regard it as such, and study to be as deserving of it as possible), to ward off wrong from innocence—to prevent the civil magistrate from ignorantly presuming to augment the infliction of Heaven, by adding bodily suffering and ignominy to bereavement of reason.

